# Three-dimensional Porous Networks of Ultra-long Electrospun SnO_2_ Nanotubes with High Photocatalytic Performance

**DOI:** 10.1007/s40820-014-0022-4

**Published:** 2014-12-10

**Authors:** Peng Zhang, Lijie Wang, Xi Zhang, Junhua Hu, Guosheng Shao

**Affiliations:** 1 School of Materials Science and Engineering, Zhengzhou University, Zhengzhou, 450002 People’s Republic of China; 2 International Joint Research Laboratory for Low-Carbon & Environmental Materials of Henan Province, Zhengzhou University, Zhengzhou, 450002 Henan People’s Republic of China; 3 Institute for Renewable Energy and Environmental Technologies, University of Bolton, Bolton, BL3 5AB UK

**Keywords:** Electrospinning, SnO_2_ nanotube, Photocurrent, Photocatalytic, Recyclability

## Abstract

Recent progress in nanoscience and nanotechnology creates new opportunities in the design of novel SnO_2_ nanomaterials for photocatalysis and photoelectrochemical. Herein, we firstly highlight a facile method to prepare three-dimensional porous networks of ultra-long SnO_2_ nanotubes through the single capillary electrospinning technique. Compared with the traditional SnO_2_ nanofibers, the as-obtained three-dimensional porous networks show enhancement of photocurrent and photocatalytic activity, which could be ascribed to its improved light-harvesting efficiency and high separation efficiency of photogenerated electron–hole pairs. Besides, the synthesis route delivered three-dimensional sheets on the basis of interwoven nanofibrous networks, which can be readily recycled for the desirable circular application of a potent photocatalyst system.

**Electronic supplementary material:**

The online version of this article (doi:10.1007/s40820-014-0022-4) contains supplementary material, which is available to authorized users.

## Introduction

It has been widely demonstrated that semiconducting materials are of great promise for energy and environmental applications such as photocatalytic water splitting for hydrogen production, dye-sensitized solar cells, and photocatalytic remediation of harmful organics from air and water [1–4]. As a typical n-type semiconductor, SnO_2_ has received great attention because of its excellent stability, nontoxicity, low-cost, and excellent optical electricity properties [5]. Especially, the exploitations of SnO_2_ for photocatalytic water splitting and photocatalytic oxidation of organic wastes have been hot topics because of its high reduction potential and low oxidation potential [6–8].

However, the practical performance of bulk SnO_2_ is far from the ideal case which has been limited by several factors such as short lifetime of the excited-state carrier (10^−12^ s), poor oxygen evolution reaction kinetics, and short hole diffusion length (2–4 nm) [9–15]. As we all know, the separation rate of photoinduced surface and volume charge carriers in a photocatalyst can be significantly increased by reducing its size to the appropriate nanoscale level and, thus, the photocatalytic activity can be enhanced. Therefore, considerable effort has been taken to synthesize photocatalysts with a small size so as to achieve high activities. However, a new disadvantage arises naturally: the recycling of the photocatalysts hinders their applications due to their small size. This has led to an unfavorable balance between the reduced charge recombination and a negative impact on recycling. Therefore, it is highly challenging but desirable to develop a direct effective approach for fabricating a new type of nanostructured photocatalyst with efficient electron–hole utilization, high specific surface areas, and favorable recycling characteristics.

Notably, electrospinning technique provides an effective approach to fabricate the three-dimensional porous supports with large surface area. In particular, it has been demonstrated successfully by different groups including ours that the three-dimensional structure composed of one-dimensional (1D) nanofibers or nanotubes catalysts with large length-to-diameter ratio can allow them to be readily separated from fluid by sedimentation [16–21]. Moreover, it is known that the structure and morphology also have a strong effect on the physical and chemical properties of photocatalysts, especially on the photocatalytic activities. Among various morphologies, hollow structures have attracted immense attention for their evidently improved performances over particles in photocatalysis and other applications. The hollow structures, especially those with tubular structures, have many useful features: (i) high surface-to-volume ratio enables it to adsorb a large amount of chemicals, (ii) hollow multi-channeled structures makes it convenient for mass transfer, (iii) the unique structure makes better use of light through multiple reflections within its hollow space.

Herein, we reported a successful attempt for the fabrication of the SnO_2_ nanotubes by the single capillary electrospinning technique. The investigation of photocatalytic ability indicated that the as-prepared composites exhibited high photocatalytic activity in the decomposition of Methyl Orange (MO). Also, free-standing sheets were readily delivered using the current processing routes, which enables circular exploitation of the materials as potent environmental catalysts.

## Experimental Section

### Fabrication of SnO_2_ Nanotubes and SnO_2_ Nanofibers

**Scheme 1:**
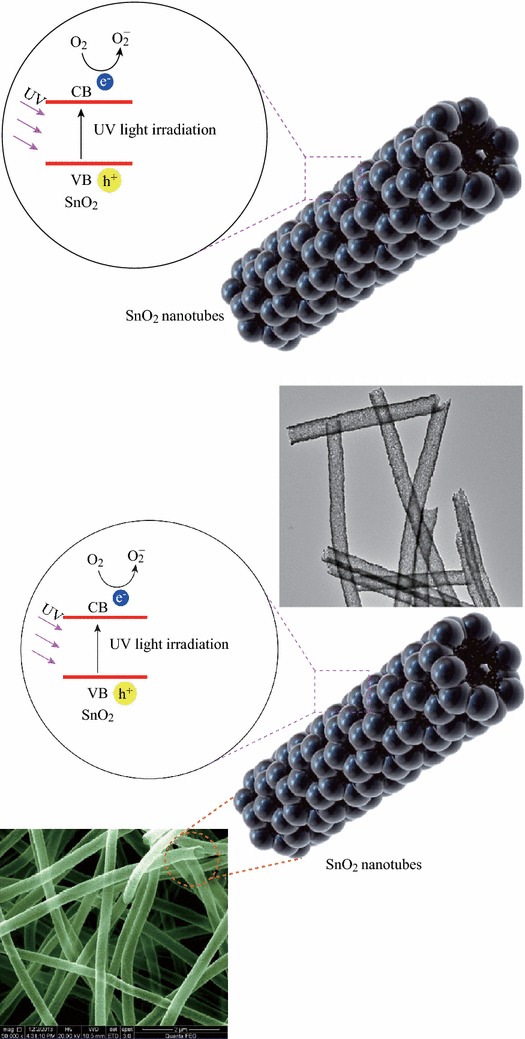
Postulate mechanism of the UV light-induced photodegradation of MO with the SnO_2_ nanotube

The fabrication of SnO_2_ NTs is schematically indicated in Scheme 1. To obtain nanotubes, two kinds of precursor solutions were prepared. Firstly, certain amounts of tin dichloride dihydrate (SnCl_2_·2H_2_O, Tianjin. Chemical Corp., China) were dissolved in a mixture of 2.2 g ethanol and 2.2 g *N,N*-dimethyl formamide by magnetic stirring for 1 h at room temperature. Secondly, 0.4 g polyvinyl pyrrolidone (PVP, Sigma Aldrich, Mw 1 300 000) was added to the resultant solution and vigorously stirred for 3 h at room temperature. The weight ratio of PVP to SnCl_2_·2H_2_O was 1.78. A high voltage of 20 kV was supplied by a direct-current power supply and the feeding rate for the precursor solution was adjusted to a constant rate of 0.3 mL h^−1^ using a syringe pump. A piece of aluminum foil was placed at 15 cm below the tip of the needle to collect the as-spun nanofibers. The process was carried out in air at room temperature. For the following thermolysis process, the as-spun nanofibers were placed in a muffle furnace and calcined at 600 °C in air for 3 h with a heating rate of 2 °C min^−1^, to remove PVP and obtain SnO_2_ nanotubes. By tuning the weight ratios of PVP to SnCl_2_·2H_2_O to 0.73, the samples of SnO_2_ nanofibers denoted as SnO_2_ NFs were fabricated.

### Characterization

The morphologies of the as-prepared nanofibers were observed by scanning electron microscopy (FESEM, JSM-7500F) at an accelerating voltage of 20 kV, transmission electron microscopy (TEM; FEI Tecnai G2 F20) at an accelerating voltage of 300 kV. X-ray diffraction (XRD) was carried out with the 2*θ* range from 20 to 80° at a scan rate of 1° min^−1^ using a D/max 2500 XRD diffractometer (Rigaku) with Cu Kα radiation (0.1541 nm). The Brunauer-Emmett-Teller (BET)-specific surface area determination was performed by N_2_ gas adsorption using an America Micromeritics ASAP 2010 surface analytical instrument.

### Photoelectrochemical Experiment

Photoelectrochemical measurements were performed using the conventional three electrode setup connected to an electrochemical station (CH Instrument 660C, Shanghai Chenhua, China). The setup had SnO_2_ NFs/FTO, and SnO_2_ NTs/FTO (effective area was 1 cm^2^, effective amount was 0.01 g) as working electrodes, and a Pt wire and an Ag/AgCl (saturated KCl) electrode were used as the counter electrode and reference electrode, respectively. The electrolyte was 0.5 M Na_2_SO_4_ aqueous solution. A 50 W high-pressure mercury lamp with main emission wavelength of 313 nm was used as the visible light source. The photocurrent response spectroscopy was carried out at a constant potential of +0.6 V to the working photoanode.

### Photocatalytic Test

**Fig. 1:**
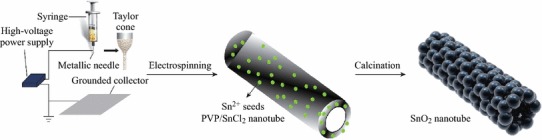
Schematic illustration of the fabrication process of the SnO_2_ nanotube

 The photocatalytic activities were evaluated by the decomposition of MO under UV light. The photoreactor was designed with an internal light source surrounded by a quartz jacket (50 W high-pressure mercury lamp with output light intensity of 1,200 mW cm^−2^ and main emission wavelength of 313 nm), where a 100 mL of the model dye (MO) solution with an initial concentration of 10 mg L^−1^ in the presence of solid catalyst (0.01 g), respectively. The solution was stirred in the dark for 30 min to obtain a good dispersion and reach adsorption–desorption equilibrium between the organic molecules and the catalyst surface. Decreases in the concentrations of dyes were analyzed by a Cary 500 UV–Vis-NIR spectrophotometer at *λ* = 553 nm. At given intervals of illumination, the samples (3 mL) of the reaction solution were taken out and centrifuged. Finally, the filtrates were analyzed. The fabrication process of SnO_2_ nanotube is illustrated in Fig. 1.

## Results and Discussion

### SEM of the As-Prepared Composite Nanofibers

**Fig. 2:**
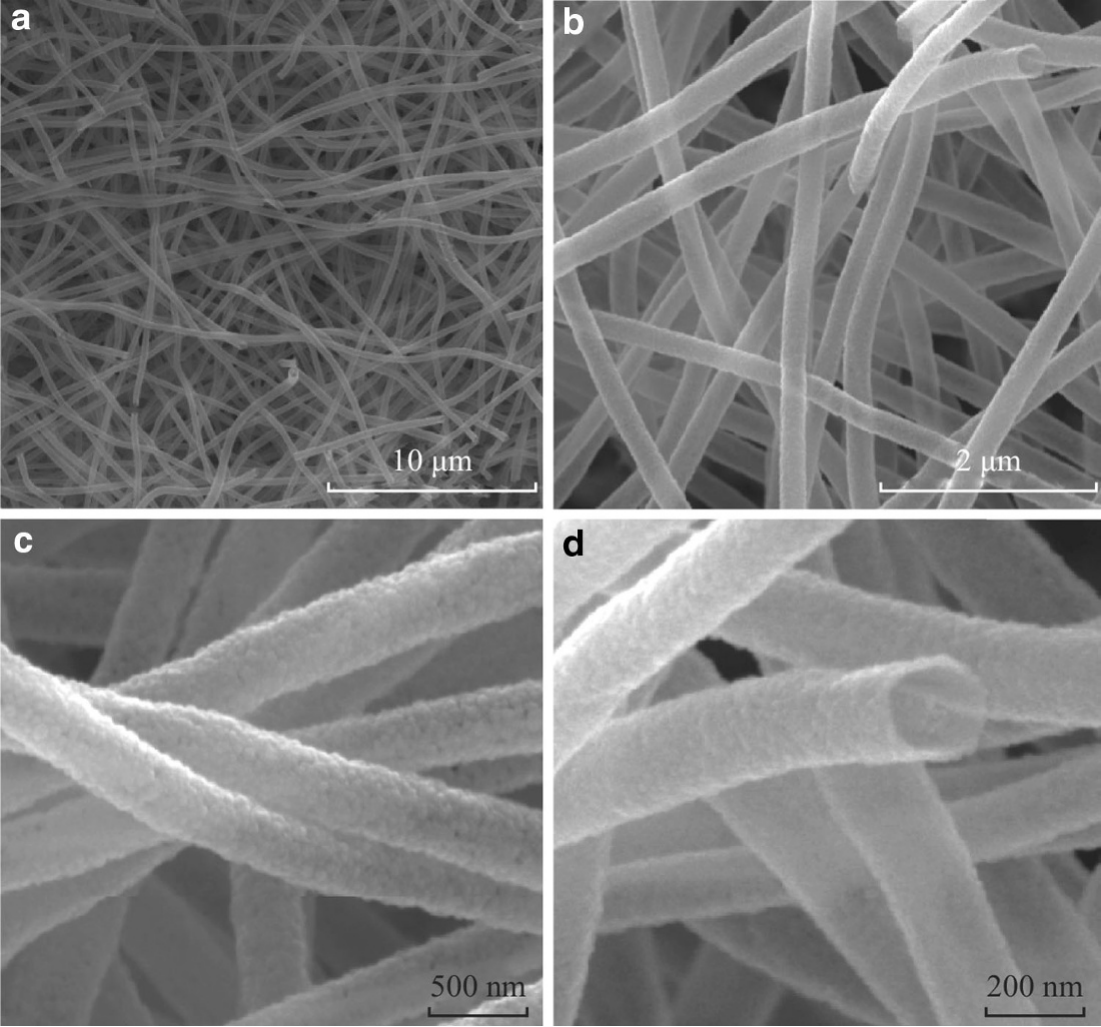
**a, b** SEM images of the SnO_2_ nanotubes; **c, d** The enlarged SEM images

The morphologies were examined by SEM and representative images are shown with the as-prepared SnO_2_ NTs. It could be seen from Fig. 2a that a free-standing three-dimensional network structure was composed of a large quantity of randomly deposited nanotubes. Figure 2b showed that the individual fiber had a high aspect ratio and a smooth surface, and the lengths of these randomly oriented nanotubes could reach several micrometers, and the diameters of those nanotubes ranged from 120 to 200 nm. Furthermore, Fig. 2c, d showed the high-magnification SEM image of Fig. 2b. It was observed that the SnO_2_ NTs had a smooth and clean surface with inner diameter of about 260 nm. In order to determine the chemical composition of the core/shell nanotube, EDX spectra (Fig. S1 in ESM) of SnO_2_ NTs in conjunction with SEM indicated that the SnO_2_ NTs were composed of Sn, O, and C, and the Pt peak came from the conductive coating when operating the SEM. It was worth pointing out that the atomic ratio of Sn to O was approximated to 1:2, which was close to the theoretical value.

### TEM of the As-Prepared Composite Nanofibers

**Fig. 3:**
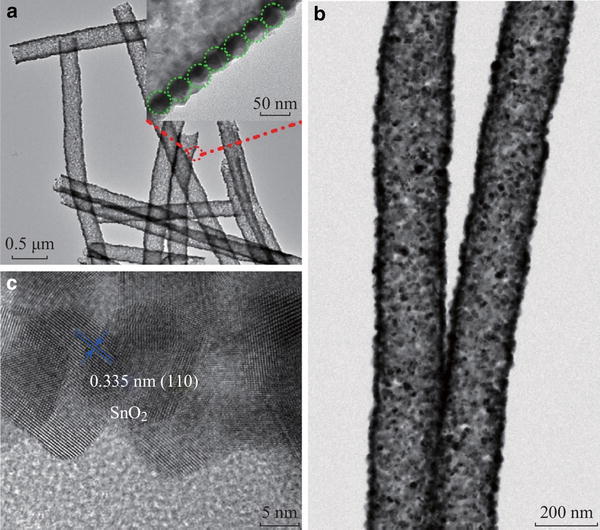
**a** TEM images of the SnO_2_ nanotube; **b** The enlarged TEM image; **c** HRTEM images of the SnO_2_ nanotube

In order to obtain more detailed information about the morphology and crystalline structure of the SnO_2_ NTs, the TEM and high resolution TEM (HRTEM) measurements were carried out. Figure 3a showed the typical TEM of the SnO_2_ NTs. It’s interesting to note that the SnO_2_ NTs were composed of nanoparticles, and each nanoparticle attached to several other nanoparticles. Furthermore, Fig. 3b showed the high-magnification TEM image of Fig. 3a. It was observed that the inner diameter of SnO_2_ NTs was about 260 nm and the wall thickness was about 45 nm. In Fig. 3c, the lattice spacing is 0.335 nm, which could be assigned to the plane [110] of SnO_2_ [22].

### XRD Patterns

**Fig. 4:**
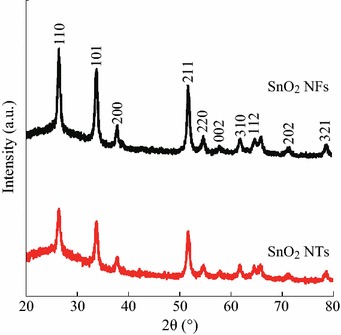
XRD images of the SnO_2_ nanofibers and SnO_2_ nanotubes

XRD analyzes were conducted to assess the structure and phase purity of the SnO_2_. As observed in Fig. 4, for pure SnO_2_ NTs and SnO_2_ NFs, ten reflection peaks appeared at 2*θ* = 26.59° (110), 33.88° (101), 38.98° (200), 51.78° (211), 54.46° (220), 53.0° (422), 57.67° (002), 62.02° (310), 71.3° (202), 78.2° (321), which were attributed to the tetragonal rutile SnO_2_ structure (space group *P42*/*mnm*, *a*_0_ = *b*_0_ = 4.737 Å, *c*_0_ = 3.186 Å, JCPDS, No. 88-0287). And, there was no evident diffraction peak attributed to Sn and SnO secondary phases, suggesting a relatively pure SnO_2_ phase could be obtained after calcination at 600 °C in air. Moreover, the average grain sizes of the products were calculated from SnO_2_ (110) peak by applying the Debye–Scherrer formula. It could be seen that the average grain size of SnO_2_ NPs was about 10 nm for both SnO_2_ NTs and SnO_2_ NFs, which was consistent with TEM observations.

### Photocatalytic Activity

**Fig. 5:**
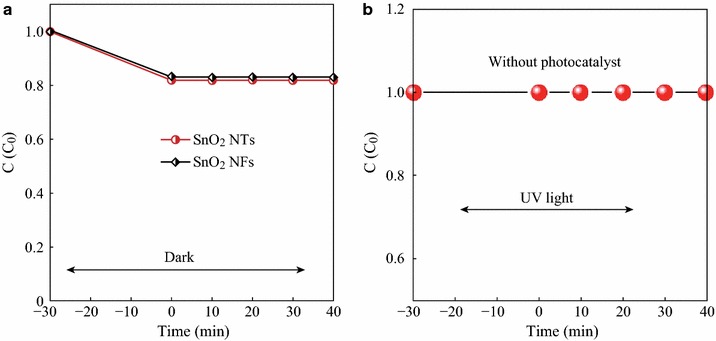
**a** Degradation profiles of MO in the *dark*; **b** Self-degradation of MO with UV light irradiation but in the absence of the nanofiber photocatalysts

To demonstrate the photoactivity of the as-obtained SnO_2_ NTs for the degradation of organic pollutants, we carried out the experiments of the photocatalytic degradation of MO as a test reaction. Furthermore, in the comparative experiments, the pure SnO_2_ NFs were used as a photocatalytic reference to understand the photocatalytic activity of the SnO_2_ NTs. As observed in Fig. 5a, b, the control experiments were performed under different conditions: (1) in the presence of photocatalysts (0.01 g) but in the dark; (2) with UV irradiation but in the absence of the photocatalysts. These control experiments revealed that there was no appreciable degradation of MO over the SnO_2_ NTs in the absence of UV light irradiation, indicating that the adsorption–desorption equilibrium of MO in the dark was established within 30 min. And, there was no appreciable degradation of MO in the absence of photocatalysts. The change in absorption spectra of MO aqueous solution showed the change of its concentration. The initial concentration (*C*_0_), the final concentration (*C*), and the degradation rate (*D*  %) had a mathematical expression as follows

1 $$D\;\%=\;\left(C_{0}-C\right)/C_{0}$$

**Fig. 6:**
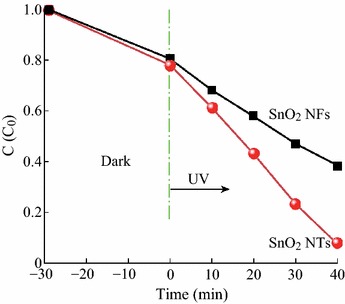
Photocatalytic activity of the samples under illumination with UV light

As shown in Fig. 6, after UV light irradiation for 40 min, the degradation efficiency of MO was about 50 and 88 % for SnO_2_ NFs and SnO_2_ NTs, respectively. Obviously, the SnO_2_ NTs showed much higher photocatalytic activities than that of the SnO_2_ NFs. What’s more, for a better comparison of the photocatalytic efficiency of the SnO_2_ NFs and SnO_2_ NTs, the kinetic analysis of degradation of MO was discussed. The kinetic linear simulation curves of the photocatalytic degradation of MO over the above catalysts showed that the above degradation reactions followed a Langmuir–Hinshelwood apparent first-order kinetics model due to the low initial concentrations of the reactants. The explanation is described below:

2 $$r=\text{d}C/\text{d}t=kKC/\left(1+KC\right)$$

where *r* is the degradation rate of the reactant (mg L min^−1^), *C* is the concentration of the reactant (mg L^−1^), *t* is the UV light irradiation time, *k* is the reaction rate constant (mg L min^−1^)), and *K* is the adsorption coefficient of the reactant (L mg^−1^). When the initial concentration (*C*_0_) is very low (*C*_0_ = 10 mg L^−1^ for MO in the present experiment), Eq. (2) can be simplified to an apparent first-order model:

3 $$-\ln C/C_{0}=kKt\;=k_{\text{app}}t$$

where *k*_app_ is the apparent first-order rate constant (min^−1^). The determined *k*_app_ values for different catalysts were summarized in Fig. S3 in ESM. The photocatalytic reactivity order was SnO_2_ NTs > SnO_2_ NFs, which was well-consistent with the activity studied above.

**Fig. 7:**
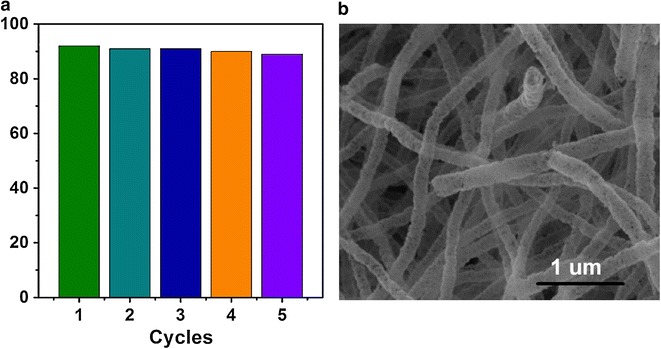
**a** Photocatalytic activity of the SnO_2_ nanotube with five times of cycling uses; **b** SEM image of the SnO_2_ nanotube after five rounds of cycling

Moreover, cycling uses as well as maintaining high photocatalytic activity was a critical issue for long-term use in practical applications of the catalyst. Consequently, two factors were needed to be considered: (i) the stability of the catalyst to maintain its high activity over time. It was known that the photocorrosion or photodissolution of catalyst might occur on the photocatalyst surface during the photocatalytic reaction. To test the stability of SnO_2_ NTs, we reused the catalyst for five times. As shown in Fig. 7a, each experiment was carried out under identical conditions in which a 100.0 mL of the model dye (MO) solution with an initial concentration of 10 mg L^−1^ in the presence of solid catalyst (0.01 g), and the photocatalytic activity of the SnO_2_ NTs remained almost unchanged in the recycling reactions. Figure 7b showed the SEM images of the SnO_2_ NTs after the fifth catalytic reaction. It could be seen that the nanostructure of SnO_2_ NTs was still very complete, clearly indicating the stability. (ii) The ease with which the catalyst could be recycled from solution. The samples were of one-dimensional nanotubes morphology and could be easily separated from an aqueous suspension. The photograph in Fig. 7 showed that the samples could be easily separated from the solution by sedimentation, probably due to the large length-to-diameter ratio of the one-dimensional SnO_2_ NTs. It was indicated that the SnO_2_ NTs displayed an efficient photoactivity for the degradation of organic pollutants under UV light irradiation and could easily be recycled for reuse.

### Postulated Photocatalytic Mechanism of the SnO_2_ NTs

It was well known that the photocatalytic activity was mainly governed by phase structure, adsorption ability, and separation efficiency of photogenerated electrons and holes. As could be seen from the XRD analysis, the crystal phase structure of the SnO_2_ NTs was nearly similar to that of SnO_2_ NFs. An adsorption experiment was performed to evaluate the adsorption ability of the SnO_2_ NFs and SnO_2_ NTs photocatalysts in the dark. As could be seen from Fig. 6, after equilibration in the dark for 30 min, 84 and 80 % of MO remained in the solution with SnO_2_ NFs and SnO_2_ NTs, respectively. Obviously, there were significant changes in the BET surface area (19.231 and 32.832 m^2^ g^−1^ for SnO_2_ NFs and SnO_2_ NTs). The enhancement of adsorption could be contributed to the increased surface area of the nanotubes. MO molecules could be adsorbed on the surface of SnO_2_ NTs until an adsorption–desorption equilibrium was reached. Compared with SnO_2_ NFs, the enhanced adsorptivity was a good supplement for the high photocatalytic activity of the SnO_2_ NTs photocatalyst.

Besides the high adsorptivity, the significant enhancement of photocatalytic activity was mainly due to the high efficiency of charge separation. According to traditional semiconductor theory shown in Scheme 1, SnO_2_ could be excited by UV light and produced photogenerated electron–hole pairs, showing photocatalytic activity. For both SnO_2_ NFs and SnO_2_ NTs, the mechanism for the photocatalytic degradation of MO in our experiment was proposed as follows (1–7):

1 $${\text{SnO}}_{2}+hv\to {\text{e}}^{-}\left({\text{SnO}}_{2}\right)\;+{\;\text{h}}^{+}\left({\text{SnO}}_{2}\right)$$

2 $${\text{e}}^{-}\left({\text{SnO}}_{2}\right)\;+{\;\text{O}}_{2}\to {\text{SnO}}_{2}+{\;\text{O}}_{2}^{-}$$

3 $${\text{O}}_{2}^{-}+{\;\text{H}}_{2}\text{O}\;\to {\;\text{HO}}_{2}\cdot \;+{\;\text{OH}}^{-}$$

4 $${\text{HO}}_{2}\cdot \;+{\;\text{H}}_{2}\text{O}\;\to {\;\text{H}}_{2}{\text{O}}_{2}+\;\text{OH}\cdot$$

5 $${\text{H}}_{2}{\text{O}}_{2}\to \;\text{2 OH}\cdot$$

6 $$\text{OH}\cdot +\text{MO}\to {\;\text{CO}}_{2}+{\;\text{H}}_{2}\text{O}$$

7 $${\text{h}}^{+}\left({\text{SnO}}_{2}\right)+\text{MO}\to {\;\text{CO}}_{2}+{\;\text{H}}_{2}\text{O}$$

As we know, the structure and morphology also have a strong effect on the efficiency of charge separation. Compared with SnO_2_ NFs, the unique nanotube structure makes better use of light through multiple reflections within its hollow space. Through enhancing the efficiency of light absorbance, the number of photoexcited charge carriers will be increased. What’s more, the underlying but probably more important advantages are the shorter bulk diffusion length produced by nanotubes with ultrathin thickness, and the hollow multi-channeled structure makes it convenient for mass transfers, which plays an important role in prolonging the lifetime of charge carriers and improve the quantum yield.

**Fig. 8:**
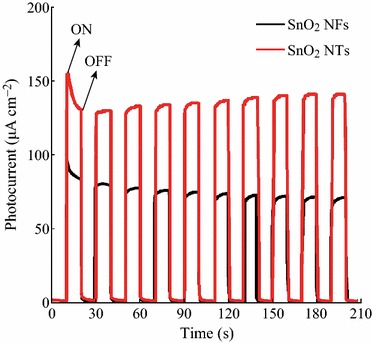
Photocurrents of SnO_2_ nanofiber and SnO_2_ nanotube electrodes under UV light irradiation *λ* = 254 nm) ([Na_2_SO_4_] 0.1 M)

Furthermore, the recombination rate of photoexcited charge carriers can be characterized by detecting the photocurrents generated [23–27]. Photoelectrochemical measurements were often used to qualitatively study the excitation and transfer of photogenerated charge carriers in photocatalysts. The transient photocurrent responses of the SnO_2_ NFs and SnO_2_ NTs were recorded for three on–off cycles under UV light irradiation and plotted in Fig. 8. As expected, SnO_2_ NTs show the higher photocurrent intensity than SnO_2_ NFs. Thus, the sample SnO_2_ NTs may achieve the more effective charge separation, which is consistent with the photocatalytic activity measurements and our above discussions.

## Conclusions

In summary, we describe herein an effective route to synthesize three-dimensional porous networks of ultra-long SnO_2_ nanotubes through the single capillary electrospinning technique. Compared with the traditional SnO_2_ nanofibers, the as-obtained three-dimensional porous networks show enhancement of photocurrent and photocatalytic activity, which could be ascribed to its improved light-harvesting efficiency and electron transport ability along the in-plane direction, and increased lifetime of photoexcited charge carriers. Besides, the synthesis route delivered three-dimensional sheets on the basis of interwoven nanofibrous networks, which can be readily recycled for the desirable circular application of a potent photocatalyst system. Notably, the free-standing 3D nanotubes network structure could improve photocatalyst’s performance of separation and reuse. Also, it is expected that the SnO_2_ NTs network will promote their industrial application as clean energy materials.

## Electronic supplementary material

Below is the link to the electronic supplementary material.

Supplementary material 1 (PDF 136 kb)
