# Supplementary material for: Three-dimensional Porous Networks of Ultra-long Electrospun SnO2 Nanotubes with High Photocatalytic Performance
Source: Nanomicro Lett. 2014 Dec 10;7:86–95. doi: 10.1007/s40820-014-0022-4 (PMC6223923; doi:10.1007/s40820-014-0022-4)
Supplement: Supplementary file 1 — Supplementary material 1 (PDF 136 kb) [file 40820_2014_22_MOESM1_ESM.pdf]

Supporting Information for

Three-dimensional Porous Networks of Ultra-long Electrospun SnO_2 Nanotubes with High Photocatalytic Performance

Peng Zhang[1,2], Lijie Wang[1,2], Xi Zhang[1,2], Changlu Shao[4] , Junhua Hu[1,2,*] and Guosheng Shao[1,2,3,*]

[1]School of Materials Science and Engineering, Zhengzhou University, Zhengzhou 450002 (People's Republic of China)
[2]International Joint Research Laboratory for Low-Carbon & Environmental Materials of Henan Province, Zhengzhou University, Zhengzhou 450002, Henan Province (People's Republic of China)
[3]Institute for Renewable Energy and Environmental Technologies, University of Bolton, Bolton BL3 5AB (UK)
[4]Center for Advanced Optoelectronic Functional Materials Research, and Key Laboratory of UV Light-Emitting Materials and Technology of Ministry of Education, Northeast Normal University (People's Republic of China)

*Corresponding author. E-mail: hujh@zzu.edu.cn; g.shao@bolton.ac.uk

Fig. S1 EDX image of the SnO_2 NTs

Fig. S2 UV-Vis diffuse reflectance spectra of the SnO_2 NTs and SnO_2 NFs

Fig. S3 Kinetic linear simulation curves of MO photocatalytic degradation with SnO_2 NTs and SnO_2 NFs

Fig. S4 The absorption spectra of SnO_2 NTs for the absorption experiment in dark

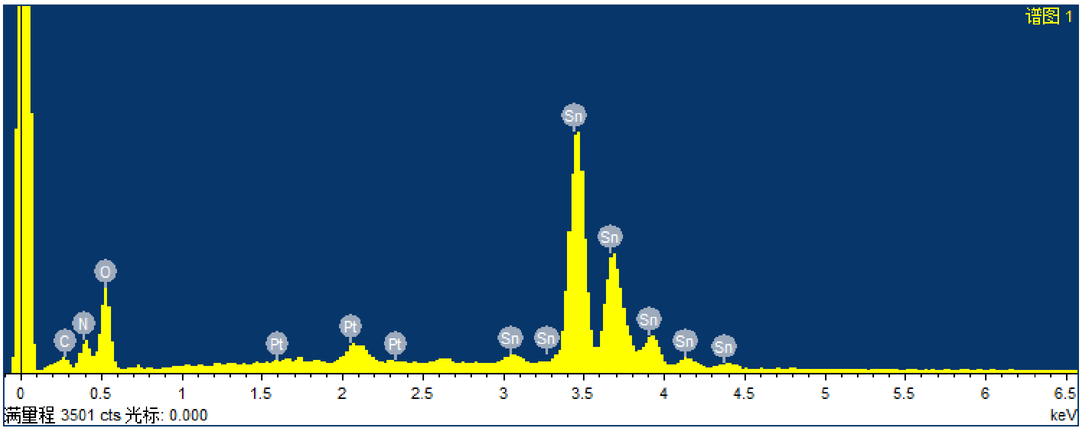

谱图 1
Sn
Sn
O
N
C
Pt
Pt
Pt
Sn
Sn
Sn
Sn
Sn
0
0.5
1
1.5
2
2.5
3
3.5
4
4.5
5
5.5
6
6.5
满里程 3501 cts 光标: 0.000
keV

Fig. S1

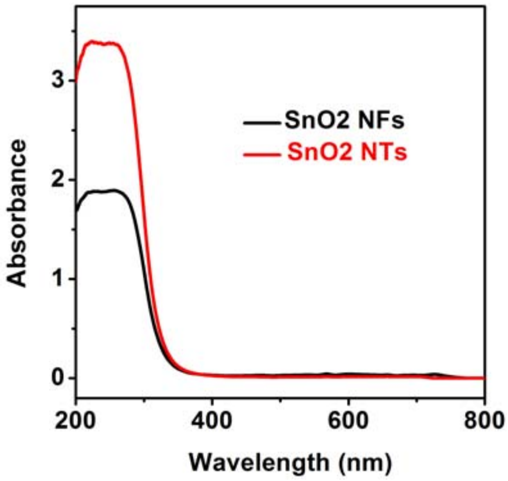

SnO2 NFs
SnO2 NTs
Absorbance
0
1
2
3
200
400
600
800
Wavelength (nm)

Fig. S2

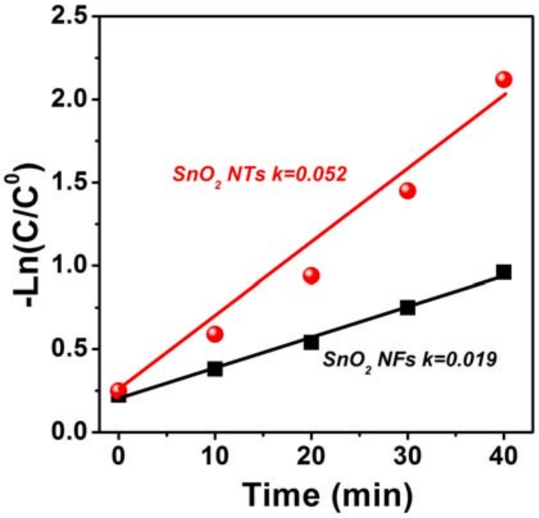

2.5
2.0
1.5
1.0
0.5
0.0
-Ln(C/C0)
SnO2 NTs k=0.052
SnO2 NFs k=0.019
0
10
20
30
40
Time (min)

Fig. S3

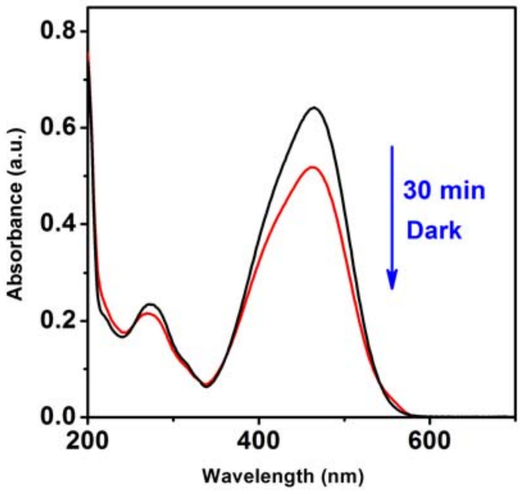

0.8
0.6
0.4
0.2
0.0
Absorbance (a.u.)
30 min
Dark
200
400
600
Wavelength (nm)

Fig. S4
